# Ibudilast, a neuroimmune modulator, reduces heavy drinking and alcohol cue-elicited neural activation: a randomized trial

**DOI:** 10.1038/s41398-021-01478-5

**Published:** 2021-06-12

**Authors:** Erica N. Grodin, Spencer Bujarski, Brandon Towns, Elizabeth Burnette, Steven Nieto, Aaron Lim, Johnny Lin, Karen Miotto, Artha Gillis, Michael R. Irwin, Christopher Evans, Lara A. Ray

**Affiliations:** 1 Department of Psychology, University of California at Los Angeles, Los Angeles, CA USA; 2 Neuroscience Interdepartmental Program, University of California at Los Angeles, Los Angeles, CA USA; 3 Institute for Digital Research and Education, University of California at Los Angeles, Los Angeles, CA USA; 4 Department of Psychiatry and Biobehavioral Sciences, University of California at Los Angeles, Los Angeles, CA USA; 5 Jane and Terry Semel Institute for Neuroscience and Human Behavior, University of California at Los Angeles, Los Angeles, CA USA; 6 Cousins Center for Psychoneuroimmunology, University of California at Los Angeles, Los Angeles, CA USA; 7 Brain Research Institute, University of California at Los Angeles, Los Angeles, CA USA

**Keywords:** Human behaviour, Psychiatric disorders

## Abstract

Ibudilast, a neuroimmune modulator which selectively inhibits phosphodiesterases (PDE)-3, -4, -10, and -11, and macrophage migration inhibitory factor (MIF), shows promise as a novel pharmacotherapy for alcohol use disorder (AUD). However, the mechanisms of action underlying ibudilast’s effects on the human brain remain largely unknown. Thus, the current study examined the efficacy of ibudilast to improve negative mood, reduce heavy drinking, and attenuate neural reward signals in individuals with AUD. Fifty-two nontreatment-seeking individuals with AUD were randomized to receive ibudilast (*n* = 24) or placebo (*n* = 28). Participants completed a 2-week daily diary study during which they filled out daily reports of their past day drinking, mood, and craving. Participants completed an functional magnetic resonance imaging (fMRI) alcohol cue-reactivity paradigm half-way through the study. Ibudilast did not have a significant effect on negative mood (β = −0.34, *p* = 0.62). However, ibudilast, relative to placebo, reduced the odds of heavy drinking across time by 45% (OR = 0.55, (95% CI: 0.30, 0.98)). Ibudilast also attenuated alcohol cue-elicited activation in the ventral striatum (VS) compared to placebo (F(1,44) = 7.36, *p* = 0.01). Alcohol cue-elicited activation in the VS predicted subsequent drinking in the ibudilast group (F(1,44) = 6.39, *p* = 0.02), such that individuals who had attenuated ventral striatal activation and took ibudilast had the fewest number of drinks per drinking day in the week following the scan. These findings extend preclinical and human laboratory studies of the utility of ibudilast to treat AUD and suggest a biobehavioral mechanism through which ibudilast acts, namely, by reducing the rewarding response to alcohol cues in the brain leading to a reduction in heavy drinking.

## Introduction

Alcohol use disorder (AUD) is a chronic relapsing disorder with a major public health impact. Over 14 million adults in the United States have an AUD [1]; however, only 8% of adults with current AUD received treatment. Only four pharmacotherapies are currently approved by the Food and Drug Administration for the treatment of AUD, and these medications are only modestly effective [2] with number needed to treat ranging from 7–144 across studies [3]. Therefore, there is a clear need to develop more efficacious treatments, particularly those with novel molecular targets [4,5,]. To that end, the modulation of neuroimmune signaling is a promising AUD treatment target.

A growing body of literature indicates that the neuroimmune system may play a critical role in the development and maintenance of AUD, termed the neuroimmune hypothesis of alcohol addiction [6]. In animal models, chronic alcohol consumption induces a neuroimmune response through the activation of microglia and increased expression of pro-inflammatory cytokines and neuronal cell death [7]. Elevated microglial markers have been identified in the postmortem brains of individuals with an AUD [8], and pro-inflammatory cytokine levels are higher in individuals with AUD compared to controls [9]. Neuroinflammation has also been implicated in mood disorders [10]. Moreover, mood states are considered to be a central feature of AUD, with a negative mood state emerging with increasing AUD severity [11]. Interactions between inflammatory pathways and the neurocircuitry activated in depression and addiction are thought to contribute to negative mood [12]. Therefore, a neuroimmune modulator may treat AUD and related negative mood symptoms through similar pathways.

Ibudilast (IBUD; also known as MN-166, previously AV411) shows promise as a novel AUD pharmacotherapy. IBUD reduced alcohol intake by 50% in two rat models, and selectively decreased drinking in alcohol-dependent mice relative to nondependent mice [13]. In a human laboratory trial, treatment with IBUD was well-tolerated and resulted in reductions in tonic craving and improvements in mood reactivity to stress and alcohol cue exposure compared to placebo [14]. IBUD is a selective phosphodiesterase (PDE) inhibitor, with preferential inhibition of PDE3A, PDE4, PDE10A, and PDE11A [15], and a macrophage migration inhibitory factor (MIF) inhibitor [16]. Both PDE4 and MIF are involved in neuroinflammatory processes through the regulation of inflammatory responses in microglia [17,18,], and PDE4B expression is upregulated after chronic alcohol exposure [19]. Therefore, IBUD is thought to reduce neuroinflammation through the inhibition of these pro-inflammatory molecules. IBUD crosses the blood–brain barrier, and is neuroprotective as it suppresses the production of pro-inflammatory cytokines and enhances the production of anti-inflammatory cytokines [20].

While IBUD is a promising AUD pharmacotherapy, its underlying mechanisms of action on the human brain remain largely unknown [21]. PDE4 is highly expressed in neuronal and non-neuronal cells including glia in brain regions associated with reward and reinforcement, including the ventral striatum (VS) [22], and PDE4 can directly regulate dopamine in the striatum in mice [23]. Functional magnetic resonance imaging (fMRI) alcohol cue-reactivity paradigms have commonly been used to evaluate if pharmacological AUD treatments alter brain activation in reward processing circuity [24,25,]. Alcohol cue-elicited reward activation is predictive of treatment response [26]; thus demonstrating that functional neuroimaging can provide mechanistic data for AUD pharmacotherapy development. This may be particularly relevant in the case of IBUD, where the mechanism of action as an AUD treatment is currently unknown, but can be hypothesized to involve the striatum, which is activated in the alcohol cue-reactivity paradigm [24,27,]. Therefore, the present study sought to investigate the efficacy of IBUD to attenuate alcohol cue-elicited VS activation in individuals with AUD.

The current study was an experimental medication trial of IBUD compared to placebo in non-treatment-seeking individuals with an AUD. To advance the development of IBUD as an AUD treatment, the present study examined the efficacy of IBUD, relative to placebo, to reduce negative mood and reduce heavy drinking (defined by the National Institute on Alcohol Abuse and Alcoholism (NIAAA) as ≥5 drinks/day for men and ≥4 drinks/day for women) over the course of 2-weeks. A micro-longitudinal design allowed for daily assessments during the course of treatment. We hypothesized that ibudilast would reduce negative mood and decrease heavy drinking over the course of the study. To investigate the neural substrates underlying IBUD’s action, the present study also examined the effect of IBUD on neural alcohol cue-reactivity. We hypothesized that ibudilast would attenuate alcohol cue-elicited activation in the VS relative to placebo. Finally, this study explored the relationship between neural alcohol cue-reactivity in the VS and drinking outcomes.

## Materials and methods

### Trial design

This was a 2-week clinical study (ClinicalTrials.gov identifier: NCT03489850) of ibudilast for negative mood improvement and drinking reduction in non-treatment-seeking individuals with an AUD. Eligible participant were randomized to ibudilast or matched placebo. Participants completed three in-person visits and daily online diary assessments to report on their drinking, craving, and mood from the previous day. This trial was approved by the Institutional review board of the University of California, Los Angeles. All study participants provided written informed consent after discussing the study medication with the study physician. Participants were enrolled and randomized in the study between July 2018 through March 2020. Data analysis were conducted from March to July 2020.

### Setting and participants

**Fig. 1:**
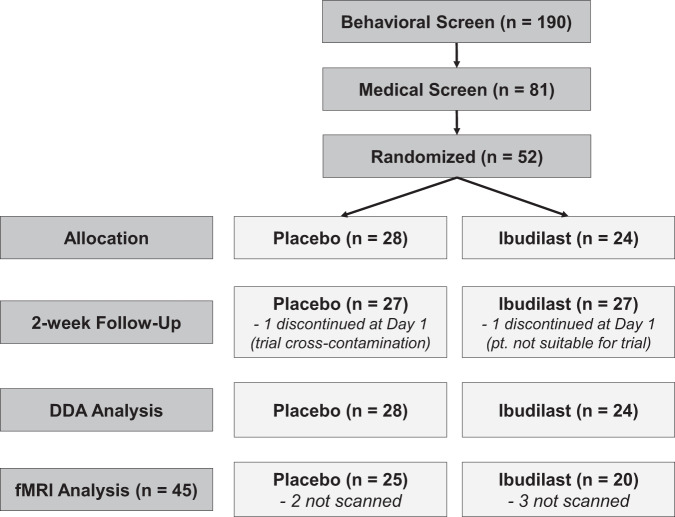
Subject flow diagram. CONSORT diagram of subject flow through the study.

This study was conducted at an outpatient research clinic in a medical center. Participants were recruited through social media and mass transit advertisements. Initial screening was conducted through telephone interview, with eligible participants invited for an in-person assessment. Eligible individuals were between 21 and 50 years old who met criteria for a current DSM-5 mild-to-severe AUD. Participants were required to drink above moderate drinking levels, as defined by the NIAAA as >14 drinks/week for men and >7 drinks/week for women, in the 30 days prior to screening. Exclusion criteria were: currently receiving or seeking treatment for AUD; past year DSM-5 diagnosis of substance use disorder (excluding alcohol or nicotine); lifetime diagnosis of schizophrenia, bipolar disorder, or any psychotic disorder; nonremovable ferromagnetic objects in body; claustrophobia; and serious head injury or prolonged period of unconsciousness (>30 min). Participants were excluded if they had a medical condition thought to interfere with safe participation and if they reported recent use of medications contraindicated with ibudilast. Women of a childbearing age had to be practicing effective contraception and could not be pregnant or nursing. See Fig. 1 for the trial enrollment flow.

### Trial procedures

A stratified randomization list was developed by a statistician and was based on sex and withdrawal-related dysphoria, a measure of AUD severity. Participants were randomized to 50 mg b.i.d. of ibudilast or placebo. MediciNova, Inc. supplied the ibudilast and placebo for the study and the UCLA Research pharmacy prepared all study medication in blister packs, which were dispensed on the randomization study visit. Participants, providers, and research staff remained blind to medication assignment throughout the study. Ibudilast was titrated as follows: 20 mg b.i.d. during days 1–2 and 50 mg b.i.d. during days 3–14. Medication compliance was monitored through pill counts at the midpoint and final study visits, as well as through self-report in the daily diary assessments. Participants were compensated up to $350 for their participation in the study ($50 for behavioral and physical eligibility screening, $150 for in-person study visits, $50 for fMRI, and $100 completion bonus if all in-person study visits and ≥80% of DDA were completed).

### Assessments

Participants completed a series of assessments for eligibility and individual differences. These measures included the Structured Clinical Interview for DSM-5 (SCID-5) [28], the Clinical Institute Withdrawal Assessment for Alcohol Scale - Revised (CIWA-Ar)[29], and the 30-day Timeline Followback Interview (TLFB) [30] for alcohol, cigarette, and cannabis. Participants also completed assessments regarding their alcohol use, including: Alcohol Use Disorder Identification Test (AUDIT) [31] and Alcohol Dependence Scale (ADS) [32], which measure severity of alcohol use problems, Penn Alcohol Craving Scale (PACS) [33] and Obsessive Compulsive Drinking Scale (OCDS) [34], which measure alcohol craving, and the Reasons for Heavy Drinking Questionnaire (RHDQ) [35] to assess withdrawal-related dysphoria, indicated by question #6: “I drink because when I stop, I feel bad (I am nervous, irritable, and I sleep poorly)”. Participants also completed measures of smoking severity (Fagerstrom Test for Nicotine Dependence (FNTD) [36]) and depressive symptomology (Beck Depression Inventory-II (BDI-II) [37]). At each in-person visit, participants were required to have a breath alcohol concentration of 0.00 g/dl and test negative on a urine toxicology screen for all drugs of abuse (except cannabis). Blood pressure and heart rate were assessed at screening and at each visit.

Participants completed three in-person study visits occurring on Day 1 (randomization), Day 8 (midpoint; neuroimaging), and Day 15 (final assessment). Randomization visits occurred on Mondays and Tuesdays to ensure that participants were at the target medication dose by the weekend. Side effects were elicited in open ended fashion and were reviewed by the study physicians (K.M. and A.G.). Adverse events were coded using the MedDRA v22.0 coding dictionary. Treatment-emergent adverse events were defined as adverse events that started after the first dose of the study drug or worsened in intensity after the first dose of study drug. Participants completed daily diary assessments, reporting on their past-day alcohol use, mood, assessed with a shortened form of the Profile of Mood States [38] (POMS), and craving, assessed through a shortened form of the Alcohol Urge Questionnaire [39] (AUQ). Participants received daily text message reminders with links to these assessments.

### Alcohol cue-reactivity task

Participants were scanned at the midpoint study visit (Day 8). Neuroimaging took place at the UCLA Center for Cognitive Neuroscience on a 3.0 T Siemens Prisma Scanner (Siemens Medical Solutions USA, Inc., Malvern, PA). Detailed neuroimaging procedures can be found in the Supplement. Participants completed a 720-s-long alcohol cue-reactivity task [40], in which they were presented with 24 pseudo-randomly interspersed blocks of alcoholic beverage images (ALC), non-alcoholic beverage images (BEV), blurred images to serve as visual controls, and a fixation cross. Each block was composed of five individual pictures of the same type, each presented for 4.8 s, for a total of 24 s. Each block was followed by a 6-s washout period during which participants reported on the urge to drink. Alcoholic beverage blocks were distributed between images of beer, wine, and liquor (two of each).

### Neuroimaging processing

Preprocessing followed conventional procedures as implemented in FMRIB Software (FSL v6.0.1 http://www.fmrib.ox.ac.uk/fsl). This included motion correction, high-pass temporal filtering (100-s cut-off), and smoothing with a 5-mm full-width, half-maximum Gaussian kernel [41]. Functional and structural data were skull-stripped to remove non-brain tissue. Each subject’s functional images were registered to their MBW, followed by their MPRAGE using affine linear transformations, and then were normalized to the Montreal Neurological Institute (MNI) 152-brain-average template through nonlinear registration [42].

A whole-brain analysis of the ALC vs. BEV contrast was conducted across groups to confirm that the paradigm activated the expected mesocorticolimbic reward circuitry (see Supplement Fig. S1 and Table S1). The mean percent signal change between the ALC and BEV blocks was then extracted from an a priori defined region of interest: bilateral VS, 6-mm-radius sphere centered at ±12 6 9 in MNI space [26], which was then reverse-registered from standard space to each participant’s anatomical image.

### Data analysis

A set of generalized estimating equations (GEEs) with compound symmetric (exchangeable) covariance structure were run in SAS 9.4 to account for repeated measures [43,44,]. GEEs were selected as the analytical method because parameter estimates are consistent even when the covariance structure is mis-specified. As such, a compound symmetric (i.e., exchangeable) covariance structure was chosen. Of note, due to missing data on all outcome and predictor variables, two participants were naturally excluded via listwise deletion for the GEE analysis.

A GEE model was first run to assess the effect of medication on negative mood. The dependent variable, negative mood (assessed via items from the POMS), was treated as continuous so a normal distribution with identity link function was chosen. A compound symmetric covariance structure was chosen to account for the repeated assessments. Independent variables for these analyses were medication (IBUD vs. PLAC), drinking day (yes vs. no), and the interaction of medication by drinking day. Sex, age, depressive symptomology (log BDI-II score [14]), and smoking status (smoker vs. nonsmoker) were examined as covariates; only significant covariates were retained in the final model to improve model clarity and ease of replication. A similar model was conducted to assess the effect of medication on craving, with the dependent variable being craving as measured by the AUQ. For both analyses, predicted means, standard errors, and 95% confidence intervals for negative mood and craving were calculated based on final models.

The dependent variables for the drinking analyses (heavy drinking and any drinking) were binary, such that 1 indicated a heavy drinking day (HDD) or drinking day and a 0 indicated no heavy drinking or drinking, respectively. A binomial distribution with logit link function was chosen to model the binary dependent variable (equivalent to a marginal logistic regression model with compound symmetric covariance structure). Since participants were not on medication at baseline (Day 0), this timepoint was excluded from the analysis. Independent variables included in the models were medication (IBUD vs. PLAC), time (measured in integer days), and the interaction of medication by time. Baseline drinking information (HDDs and drinking days, respectively) were also included in the model as a control. As above, sex, age, depressive symptomology (log BDI-II score), and smoking status (smoker vs. nonsmoker) were examined as covariates; only significant covariates were retained in the final model to improve model clarity and ease of replication. For both analyses, predicted probabilities, standard errors, and 95% confidence intervals for heavy drinking and any drinking were calculated based on final models.

A general linear model was used to evaluate the effect of medication on VS activation. The dependent variable was VS percent signal change between ALC and BEV blocks. Medication (IBUD vs. PLAC) was the independent variable. Age, sex, depressive symptomology (log BDI-II), and smoking status (smoker vs. nonsmoker) were examined as covariates; only significant covariates were retained in the final model. Finally, to evaluate if VS activation interacted with medication in predicting drinking in the week following the scan, a between-subject factor for VS activation (median split across medication [26]; median = 0.07) was added to the model, along with a medication by VS activation split interaction. The dependent variable was drinks per drinking day in the last week of the study. Baseline drinks per drinking day were included as an additional covariate for this analysis.

## Results

Screening and randomization of participants are summarized in Fig. 1. A total of 52 individuals were randomized in the trial, 50 completed the study, and 45 provided usable neuroimaging data (ibudilast, *n* = 20; placebo, *n* = 25). Participant demographics, drinking, and mood characteristics are presented in Table 1. There were no significant differences between groups on any baseline characteristics. There were no significant differences in adverse events across the groups across symptom categories (*p*’*s* > 0.40; see Supplement Table S2 for details). Overall medication compliance was high (98.71% compliance via self-report from the DDA and 97.05% compliance via pill count assessed at each in-person visit). There was no significant difference between groups on medication adherence (*p* > 0.39, see Supplement Table S3 for details).

**Table 1:** Sample characteristics at baseline by treatment condition.

Variable	Ibudilast (*n* = 24)	Placebo (*n* = 28)
Demographics		
Age	34.46 ± 9.24	31.07 ± 7.81
Sex, No. (%)		
Male	16 (66.7%)	18 (64.3%)
Female	8 (33.3%)	10 (35.7%)
Race/Ethnicity, No (%)		
White	17 (70.8%)	12 (42.9%)
African American	5 (20.8%)	2 (7.1%)
Asian	0 (0%)	5 (17.9%)
Pacific Islander	0 (0%)	1 (3.6%)
Mixed Race	1 (4.2%)	5 (17.9%)
Another Race	1 (4.2%)	3 (10.7%)
Hispanic or Latino	5 (20.8%)	7 (25%)
Years of Education	15.21 ± 2.64	15.21 ± 1.75
Drinking characteristics		
Withdrawal-related dysphoria (Y/N)^a^	9/15	11/17
AUD symptom count	5.29 ± 2.37	4.86 ± 2.27
AUDIT total score	16.38 ± 5.90	16.71 ± 6.42
ADS total score	13.00 ± 6.10	12.07 ± 7.01
PACS total score	12.79 ± 1 5.14	12.11 ± 7.04
OCDS total	14.54 ± 6.05	13.93 ± 8.07
RHDQ – reinforcing	23.29 ± 3.51	22.82 ± 4.88
RHDQ – normalizing	9.67 ± 7.1	8.29 ± 7.34
Total drinks (30 days)^b^	122.89 ± 64.58	114.119 ± 108.72
Drinking days (30 days)^b^	22.21 ± 6.87	20.25 ± 6.51
Drinks per day (30 days)^b^	4.10 ± 2.15	3.81 ± 3.62
Drinks per drinking days (30 days)^b^	5.70 ± 2.58	5.34 ± 3.57
Heavy drinking days (30 days)^b^	10.79 ± 8.29	8.68 ± 8.04
Cigarette and cannabis characteristics		
Cigarette smokers (%)	11 (45.8%)	14 (50%)
FTND score	2.82 ± 2.82	1.07 ± 1.54
Total cigarettes (30 days)^b^	52.28 ± 79.85	133.07 ± 205.78
Cigarettes per day (30 days)^b^	7.39 ± 8.70	5.06 ± 6.76
THC + urine (Y/N)	7/17	8/20
Cannabis days (30 days)^b^	11.38 ± 9.99 (*n* = 13)	8.15 ± 8.24(*n* = 13)
Other characteristics		
BDI-II total score	12.42 ± 8.47	8.64 ± 7.82

Data were presented as mean ± standard devation.

*AUDIT* alcohol use disorder identification test, *ADS* alcohol dependence scale, *PACS* penn alcohol craving scale, *OCDS* obsessive compulsive drinking scale, *RHDQ* reasons for heavy drinking questionnaire, *FTND* Fagerstrom test for nicotine dependence, *BDI-II* Beck depression inventory-II.

^a^Assessed by response to question #6 on the RHDQ.

^b^Assessed by Timeline Followback (TLFB) interview for the past 30 days.

### Medication effects on negative mood

There were no significant main effects of medication or medication by drinking interactions on daily mood (see Table 2). The predicted means of negative mood for ibudilast and placebo on drinking and non-drinking days were as follows: ibudilast non-drinking day: 2.95 ± 0.54 (95% CI: 1.89, 4.01); ibudilast drinking day: 2.87 ± 0.59 (95% CI: 1.76, 3.99); placebo non-drinking day: 2.40 ± 0.42 (95% CI: 1.57, 3.23); and placebo drinking day: 2.53 ± 0.39 (95% CI: 1.77, 3.30).

**Table 2:** Effect of ibudilast on heavy drinking and craving.

Model and predictor variables	Parameter estimate	SE	95% confidence limits				Odds ratio	95% confidence limits	
			LL	UL	Z	P		LL	UL
Negative mood (POMS)									
Medication (IBUD)	0.34	0.69	−1.01	1.69	0.49	0.62			
Drinking day (no)	−0.13	0.18	−0.48	0.22	−0.74	0.46			
Med X drinking day	0.21	0.49	−0.76	−0.48	0.42	9.67			
Heavy drinking									
**Medication (IBUD)**	−**0.60**	**0.30**	**−1.19**	**−0.02**	**−2.02**	**0.04**	**0.55**	**0.30**	**0.98**
Time	0.003	0.02	−0.04	0.04	0.16	0.87	1.00	0.96	1.04
**Smoking status (nonsmoker)**	**−0.87**	**0.32**	**−1.50**	**−0.24**	**−2.71**	**0.007**	**0.42**	**0.22**	**0.79**
**Baseline HDD**	**0.08**	0.03	**0.03**	**0.13**	**2.99**	**0.003**	**1.08**	**1.03**	**1.14**
Craving (AUQ)									
Medication (IBUD)	−0.16	0.76	−1.62	1.30	−0.21	0.83			
**Drinking day (no)**	**−1.06**	**0.28**	**−1.61**	**−0.50**	**−3.75**	**<0.001**			
*Med X Drinking Day*	*−0.90*	*0.49*	*−1.86*	*0.06*	*−1.84*	*0.07*			

Bolded typeface indicates a significant effect. Italic typeface indicates a trend-level effect.

### Medication effects on HDDs, any drinking days, and craving

**Fig. 2:**
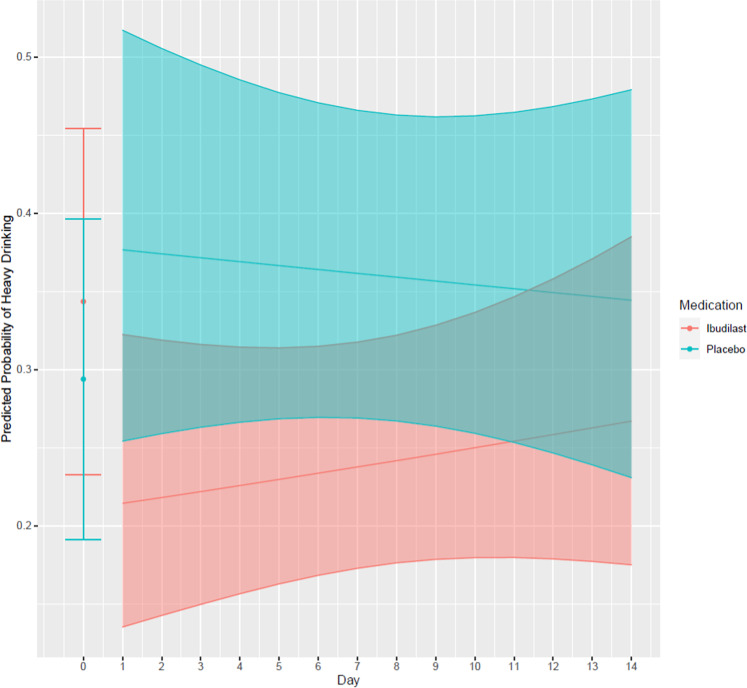
Effect of ibudilast on heavy drinking days. Ibudilast reduced heavy drinking across time as compared to placebo, controlling for baseline heavy drinking and smoking status. Baseline percent heavy drinking is indicated as Day 0 for each medication group. Heavy drinking is presented as predicted probability of heavy drinking for each day per medication condition in the micro-longitudinal study. The shaded regions are the 95% confidence intervals surrounding each prediction.

The final model for HDD included time, medication, smoking status, and baseline heavy drinking. IBUD significantly reduced HDD compared to placebo (see Fig. 2 and Table 2). The odds ratio of IBUD compared to PLAC was 0.55 (95% CI:0.30, 0.98), which indicates a 45% reduction in the odds of heavy drinking across time. The predicted probability (in percent) of heavy drinking in the ibudilast group was 24.16 ± 4.05% (95% CI: 17.12, 32.94), whereas the predicted probability of heavy drinking in the placebo group was 36.80 ± 4.54% (95% CI: 28.43, 46.05). There was no significant effect of time, nor a significant medication by time interaction.

The final model for any drinking days included time, medication, and baseline drinking days. The effect of medication on drinking days was not significant (see Supplement Table S4; OR = 0.83, (95% CI: 0.47, 1.48)) due primarily to the high standard error in estimates. The predicted probability (in percent) of any drinking in the ibudilast group was 59.25 ± 5.78% (95% CI: 47.62, 69.92), whereas the predicted probability of any drinking in the placebo group was 63.63 ± 4.40% (95% CI: 54.65, 71.75).

The final model for craving included medication, drinking day (yes/no), and medication by drinking day interaction. There was a trend towards an interaction between medication and drinking on craving (*p* = 0.07; see Table 2), such that on non-drinking days, IBUD reduced craving compared to placebo (Z = 2.27, *p* = 0.02; see Supplementary Fig. S2). The predicted means of craving for ibudilast and placebo on drinking and non-drinking days were as follows: ibudilast non-drinking day: 2.48 ± 0.42 (95% CI: 1.66, 3.29); ibudilast drinking day: 4.44 ± 0.54 (95% CI: 3.38, 5.50); placebo non-drinking day: 3.54 ± 0.48 (95% CI: 2.59, 4.49); and placebo drinking day: 4.60 ± 0.52 (95% CI: 3.59, 5.61).

### Medication effects on alcohol cue-reactivity

**Fig. 3:**
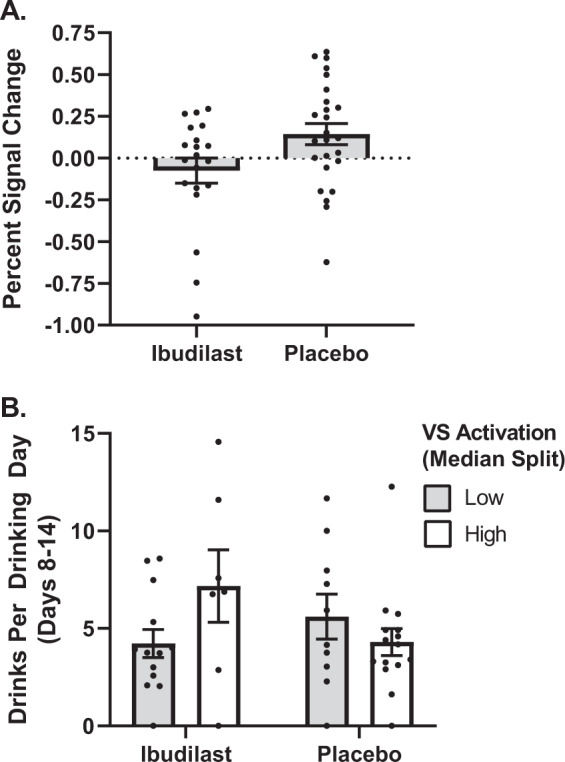
Effect of ibudilast on alcohol cue-induced activation in the ventral striatum. **A** IBUD attenuated the percent signal change to alcohol vs. beverage cues as compared to placebo (*p* = 0.01). **B** Medication by ventral striatal activation interaction. There was a significant medication by striatal activation (median split) interaction (*p* = 0.01). Individuals treated with IBUD who showed attenuated ventral striatal activation to alcohol cues had the fewest drinks per drinking day in the week following the fMRI scan.

The alcohol cue-reactivity paradigm elicited the expected mesocorticolimbic brain activation across participants (see Supplement Fig. S1 and Table S1). IBUD significantly attenuated bilateral ventral striatal activation to alcohol cues, (F(1,44) = 7.36, *p* = 0.01; Fig. 3A). There was also a significant effect of sex on percent signal change (F(1,44) = 6.39, *p* = 0.02), such that men had higher activation than women; however the interaction between medication and sex was not significant (*p* = 0.26). There were no significant effects of age, smoking status, or depressive symptomatology on VS activation.

### Prediction of drinking outcomes from medication and VS activation

There was a significant interaction between medication and activation in the VS on subsequent drinking (drinks per drinking day) (F(1,40) = 6.85, *p* = 0.01), such that individuals who had decreased activation to ALC vs. BEV and were treated with IBUD had the fewest number of drinks per drinking day in the week following the scan (Fig. 3B). There were no significant effects of age, sex, smoking status, or depressive symptomatology on subsequent drinking.

## Discussion

This was the first study to evaluate the effects of ibudilast, a neuroimmune modulator, on mood and drinking outcomes in a clinical sample with AUD. Contrary to our hypothesis, ibudilast did not have a significant effect on negative mood on drinking or non-drinking days. However, in support of our hypotheses, ibudilast significantly reduced the probability of heavy drinking compared to placebo. Ibudilast also significantly attenuated alcohol cue-elicited activation in the bilateral VS. Furthermore, exploratory analyses indicated that ventral striatal activation to alcohol cues was predictive of drinking in the week following the neuroimaging scan. These results suggest a biobehavioral mechanism through which ibudilast acts, namely, by reducing the rewarding response to alcohol cues in the brain leading to a reduction in heavy drinking per se.

Unexpectedly, this study did not find support for an effect of ibudilast on negative mood or a moderating effect of baseline depressive symptomology on medication response. This contrasts with previous findings from our lab in which ibudilast improved mood response to stress and alcohol cues [14]. The current study differs from the previous study in several important methodological variables including using a between-subjects instead of a crossover design and the use of a daily-diary mood reporting approach compared to tightly controlled human laboratory experimental paradigms. Furthermore, the current study did not directly evaluate the effect of drinking on mood, which would be more comparable to the findings reported previously. Additionally, this study recruited individuals with mild-to-severe AUD. Negative mood states and negative reinforcement driven drinking may only occur at more severe presentations of AUD [11]; therefore, the present study may have been underpowered to identify medication effects on negative mood symptoms.

Regarding the drinking outcomes in this study, IBUD significantly reduced the probability of heavy drinking compared to placebo. Specifically, individuals treated with IBUD were 45.3% less likely (OR = 0.547) to drink heavily compared to individuals treated with placebo. This resulted in a 24% predicted probability of heavy drinking over the course of the study in the ibudilast group, compared with a 37% predicted probability in the placebo group. Of note, there were no significant differences in AE’s between groups, indicating that this reduction was not due to increased side effects, including nausea, in the IBUD group. There was not a significant effect of IBUD on the probability of overall drinking compared to placebo. While nonsignificant, the effect of IBUD for any drinking days was in the expected direction, such that individuals on IBUD were 16.9% less likely (OR = 0.831) to engage in any drinking relative to placebo, but high variability in the prediction prevented conclusive statistical findings. This nonsignificant effect may not be surprising, as the study sample was comprised of non-treatment-seekers and therefore not motivated to abstain from drinking altogether. Rather, participants treated with IBUD reduced their heavy drinking, which produces a harm reduction benefit, particularly for those with a mild-to-moderate AUD [45,46,]. This finding is also consistent with preclinical studies, where treatment with ibudilast reduced ethanol intake by 50% under maintenance conditions [13]. Importantly, the drinking results combined with the AE reports indicate that ibudilast is a safe medication for individuals who are still drinking and may want to reduce their drinking. IBUD also reduced craving on non-drinking days, at trend level, as compared to placebo. This effect supports our previous finding of a reduction in tonic craving under ibudilast during a week-long human laboratory study during which participants were instructed not to drink [14].

This study also examined a potential biobehavioral mechanism underlying IBUD’s action using an fMRI alcohol cue-reactivity paradigm. IBUD attenuated alcohol cue-elicited reward activation in the VS compared to placebo. PDE4 and PDE10 are highly expressed in the striatum and negatively regulate dopaminergic signaling [47]. Thus, inhibition of these PDEs through IBUD may reduce striatal excitability to alcohol cues. In rats IBUD reduced morphine-induced nucleus accumbens dopamine release [48]. Moreover, IBUD has been shown to enhance the production of neurotrophic factors, including glia-derived neurotrophic factor (GDNF) [20], which is a critical survival factor for dopamine neurons [49]. Preclinical findings indicate that infusion of GDNF normalizes dopamine levels in the ventral tegmental area and the VS and reduces alcohol seeking and alcohol consumption [50]. In humans with AUD, GDNF levels are reduced in blood serum samples [51]. Furthermore, in individuals with AUD, presentation of alcohol cues reduced interleukin-10, an anti-inflammatory cytokine, and the level of reduction was correlated with increased alcohol craving [52]. Thus, though the underlying molecular mechanism is still unknown, this finding indicates that ibudilast may normalize the dopaminergic response to alcohol cues in individuals with AUD.

This study has several strengths and limitations which should be considered when interpreting the results. Study strengths include the use of daily diary reporting, which captures real-world drinking and minimizes recall bias, and the combination of neurobiological (fMRI) with behavioral and self-report methodologies. However, this study recruited a non-treatment-seeking sample; therefore, these findings may not generalize to a treatment-seeking sample with AUD (see [53]). An ongoing randomized controlled trial of IBUD in treatment-seeking individuals with AUD (NCT03594435) will address this open question. Relatedly, this study recruited individuals with mild-to-severe AUD, which may not be representative of clinical samples. This limitation may have impacted our ability to detect medication effects that require a pathology associated with more severe AUD, which is particularly relevant for negative mood and withdrawal states. Furthermore, participants were required to have a 0.00 g/dl breath alcohol reading for each in-person visit. This requirement was to ensure participant safety; however, it may have artificially reduced drinking on in-person study visit days. Of note, in the daily diary assessment, participants reported on their past day drinking for the full day and were able to begin drinking when they returned home after the study visit. Additionally, the sample size for this experimental study was modest, particularly for the fMRI outcomes. This limited our ability to conduct additional, whole-brain analyses which are necessary to fully elucidate the neural mechanism of ibudilast. Finally, this study did not include a fixed-dose alcohol challenge to evaluate the safety and efficacy of ibudilast in combination with alcohol and to replicate our previous work. However, given that our sample did report drinking while taking ibudilast, we believe that ibudilast can be safely taken with alcohol with limited side effects.

In conclusion, this is the first combined clinical and neuroimaging study of ibudilast (50 mg b.i.d.), a neuroimmune modulator, to treat AUD. Ibudilast did not improve negative mood on drinking or non-drinking days, indicating that its mechanism of action may be non-mood dependent in non-treatment-seeking individuals. Ibudilast reduced the probability of HDDs over 2 weeks for non-treatment-seeking individuals relative to placebo. Ibudilast also attenuated alcohol cue-elicited activation in the VS, potentially through a dopaminergic-related mechanism. This is a critical proof-of-mechanism whereby modulation of neuroimmune signaling via ibudilast reduced the incentive salience of alcohol cues in the brain. Exploratory analyses indicated that ventral striatal activation to alcohol cues was predictive of subsequent drinking in the ibudilast group, such that individuals who had attenuated ventral striatal activation and were treated with ibudilast had the fewest number of drinks per drinking day in the week following the scan. Overall, these findings extend preclinical [13] and human laboratory [14] demonstrations of the efficacy of ibudilast for the treatment of AUD and suggest a potential biobehavioral mechanism through which ibudilast acts. This study also demonstrates that ibudilast has a favorable side effect profile, even when combined with alcohol. These findings also provide novel insights into the role of neuroimmune modulation in AUD, including its effects on neural and behavioral outcomes of high clinical significance.

## Supplementary information

Supplementary Material for Ibudilast, A Neuroimmune Modulator, Reduces Heavy Drinking and Alcohol Cue-Elicited Neural Activation: A Randomized Trial

## References

[1] Substance Abuse and Mental Health Servies Administration (2018). Key substance use and mental health indicators in the United States: Results from the 2017 National Survey on Drug Use and Health (HHS Publication No. SMA 18-5068, NSDUH Series H-53). Rockville, MD: Center for Behavioral Health Statistics and Quality. Substance Abuse and Mental Health Services Administration. https://www samhsa gov/data (2019).

[2] Ray LA (2019). State-of-the-art behavioral and pharmacological treatments for alcohol use disorder. Am J Drug Alcohol Abus..

[3] Falk DE (2019). Evaluation of drinking risk levels as outcomes in alcohol pharmacotherapy trials: a secondary analysis of 3 randomized clinical trials. JAMA Psychiatry..

[4] Litten RZ (2012). Medications development to treat alcohol dependence: a vision for the next decade. Addiction Biol..

[5] Litten RZ, Falk DE, Ryan ML, Fertig JB (2016). Discovery, development, and adoption of medications to treat alcohol use disorder: goals for the phases of medications development. Alcohol Clin Exp Res..

[6] Mayfield J, Harris RA (2017). The neuroimmune basis of excessive alcohol consumption. Neuropsychopharmacology..

[7] Crews FT (2015). Neuroimmune function and the consequences of alcohol exposure. Alcohol Res..

[8] He J, Crews FT (2008). Increased MCP-1 and microglia in various regions of the human alcoholic brain. Exp Neurol..

[9] Leclercq S (2012). Role of intestinal permeability and inflammation in the biological and behavioral control of alcohol-dependent subjects. Brain Behav Immun..

[10] Rosenblat JD, Cha DS, Mansur RB, McIntyre RS (2014). Inflamed moods: a review of the interactions between inflammation and mood disorders. Prog. NeuroPsychopharmacol Biol Psychiatry..

[11] Koob GF, Volkow ND (2010). Neurocircuitry of addiction. Neuropsychopharmacology..

[12] Coleman LG, Crews FT. Innate immune signaling and alcohol use disorders. In: Grant KA, Lovinger DM, editors. The neuropharmacology of alcohol. Cham: Springer International Publishing; 2018. p. 369–96.

[13] Bell RL (2015). Ibudilast reduces alcohol drinking in multiple animal models of alcohol dependence. Addiction Biol..

[14] Ray LA (2017). Development of the neuroimmune modulator ibudilast for the treatment of alcoholism: a randomized, placebo-controlled, human laboratory trial. Neuropsychopharmacology.

[15] Gibson LC (2006). The inhibitory profile of Ibudilast against the human phosphodiesterase enzyme family. Eur J Pharmacol.

[16] Cho Y (2010). Allosteric inhibition of macrophage migration inhibitory factor revealed by ibudilast. Proc Natl Acad Sci USA..

[17] Hertz AL (2009). Elevated cyclic AMP and PDE4 inhibition induce chemokine expression in human monocyte-derived macrophages. Proc Natl Acad Sci USA..

[18] Mitchell RA (2002). Macrophage migration inhibitory factor (MIF) sustains macrophage proinflammatory function by inhibiting p53: regulatory role in the innate immune response. Proc Natl Acad Sci USA.

[19] Gobejishvili L, Barve S, Joshi-Barve S, McClain C (2008). Enhanced PDE4B expression augments LPS-inducible TNF expression in ethanol-primed monocytes: relevance to alcoholic liver disease. Am J Physiol Gastrointest Liver Physiol..

[20] Mizuno T (2004). Neuroprotective role of phosphodiesterase inhibitor ibudilast on neuronal cell death induced by activated microglia. Neuropharmacology..

[21] Fox RJ (2018). Phase 2 trial of ibudilast in progressive multiple sclerosis. N Engl J Med..

[22] Pérez-Torres S (2000). Phosphodiesterase type 4 isozymes expression in human brain examined by in situ hybridization histochemistry and [3H] rolipram binding autoradiography: comparison with monkey and rat brain. J Chem Neuroanat..

[23] Liu X, Zhong P, Vickstrom C, Li Y, Liu QS (2017). PDE4 inhibition restores the balance between excitation and inhibition in VTA dopamine neurons disrupted by repeated in vivo cocaine exposure. Neuropsychopharmacology..

[24] Grodin EN, Ray LA (2019). The use of functional magnetic resonance imaging to test pharmacotherapies for alcohol use disorder: a systematic review. Alcohol Clin Exp Res..

[25] Courtney KE, Schacht JP, Hutchison K, Roche DJ, Ray LA (2016). Neural substrates of cue reactivity: association with treatment outcomes and relapse. Addiction Biol..

[26] Schacht JP (2017). Predictors of naltrexone response in a randomized trial: reward-related brain activation, OPRM1 genotype, and smoking status. Neuropsychopharmacology..

[27] Schacht JP, Anton RF, Myrick H (2013). Functional neuroimaging studies of alcohol cue reactivity: a quantitative meta‐analysis and systematic review. Addiction Biol..

[28] First M, Williams J, Karg R, Spitzer R. Structured clinical interview for DSM-5—Research version (SCID-5 for DSM-5, research version; SCID-5-RV). Arlington, VA: American Psychiatric Association; 2015.

[29] Sullivan JT, Sykora K, Schneiderman J, Naranjo CA, Sellers EM (1989). Assessment of alcohol withdrawal: the revised clinical institute withdrawal assessment for alcohol scale (CIWA‐Ar). Br J Addiction..

[30] Sobell LC, Sobell MB. Timeline follow-back. Measuring alcohol consumption. Springer; 1992.

[31] Saunders JB, Aasland OG, Babor TF, De la Fuente JR, Grant M (1993). Development of the alcohol use disorders identification test (AUDIT): WHO collaborative project on early detection of persons with harmful alcohol consumption‐II. Addiction..

[32] Skinner HA, Horn JL. Alcohol dependence scale (ADS): user’s guide. Addiction Research Foundation; 1984.

[33] Flannery B, Volpicelli J, Pettinati H (1999). Psychometric properties of the Penn alcohol craving scale. Alcohol Clin Exp Res..

[34] Anton RF, Moak DH, Latham P (1995). The obsessive compulsive drinking scale: a self‐rated instrument for the quantification of thoughts about alcohol and drinking behavior. Alcohol Clin Exp Res..

[35] Adams ZW, Schacht JP, Randall P, Anton RF (2016). The reasons for heavy drinking questionnaire: factor structure and validity in alcohol-dependent adults involved in clinical trials. J Stud Alcohol Drugs..

[36] Heatherton TF, Kozlowski LT, Frecker RC, Fagerstrom KO (1991). The Fagerström test for nicotine dependence: a revision of the Fagerstrom Tolerance Questionnaire. Br J Addiction.

[37] Beck AT, Steer RA, Brown GK (1996). Beck depression inventory-II. San. Antonio.

[38] Curran SL, Andrykowski MA, Studts JL (1995). Short form of the profile of mood states (POMS-SF): psychometric information. Psychological Assess..

[39] Bohn MJ, Krahn DD, Staehler BA (1995). Development and initial validation of a measure of drinking urges in abstinent alcoholics. Alcohol Clin Exp Res..

[40] Schacht JP (2011). Stability of fMRI striatal response to alcohol cues: a hierarchical linear modeling approach. Neuroimage.

[41] Jenkinson M, Bannister P, Brady M, Smith S (2002). Improved optimization for the robust and accurate linear registration and motion correction of brain images. Neuroimage.

[42] Andersson JL, Jenkinson M, Smith S. Non-linear registration aka spatial normalisation FMRIB Technial Report TR07JA2. FMRIB Analysis Group of the University of Oxford; 2007:1–22.

[43] Liang K-Y, Zeger SL (1986). Longitudinal data analysis using generalized linear models. Biometrika.

[44] Ziegler A. Generalized estimating equations. Springer Science & Business Media; 2011.

[45] Mann K, Aubin H-J, Charlet K, Witkiewitz K (2017). Can reduced drinking be a viable goal for alcohol dependent patients?. World Psychiatry.

[46] Hasin DS (2017). Change in non-abstinent WHO drinking risk levels and alcohol dependence: a 3 year follow-up study in the US general population. Lancet Psychiatry.

[47] Ramirez D, Smith SM (2014). Regulation of dopamine signaling in the striatum by phosphodiesterase inhibitors: novel therapeutics to treat neurological and psychiatric disorders. Cent Nerv Syst Agents Med Chem..

[48] Bland ST, Hutchinson MR, Maier SF, Watkins LR, Johnson KW (2009). The glial activation inhibitor AV411 reduces morphine-induced nucleus accumbens dopamine release. Brain Behav Immun..

[49] Airaksinen MS, Saarma M (2002). The GDNF family: signalling, biological functions and therapeutic value. Nat Rev Neurosci..

[50] Barak S, Carnicella S, Yowell QV, Ron D (2011). Glial cell line-derived neurotrophic factor reverses alcohol-induced allostasis of the mesolimbic dopaminergic system: implications for alcohol reward and seeking. J Neurosci..

[51] Heberlein A (2010). BDNF and GDNF serum levels in alcohol-dependent patients during withdrawal. Prog NeuroPsychopharmacol Biol Psychiatry.

[52] Fox HC, Milivojevic V, Angarita GA, Stowe R, Sinha R (2017). Peripheral immune system suppression in early abstinent alcohol-dependent individuals: Links to stress and cue-related craving. J Psychopharmacol..

[53] Ray LA, Bujarski S, Yardley MM, Roche DJ, Hartwell EE (2017). Differences between treatment-seeking and non-treatment-seeking participants in medication studies for alcoholism: do they matter?. Am J Drug Alcohol Abus..

