# Supplementary material for: Ibudilast, a neuroimmune modulator, reduces heavy drinking and alcohol cue-elicited neural activation: a randomized trial
Source: Transl Psychiatry. 2021 Jun 12;11:355. doi: 10.1038/s41398-021-01478-5 (PMC8197758; doi:10.1038/s41398-021-01478-5)

**Supplementary Material for**

Ibudilast, A Neuroimmune Modulator, Reduces Heavy Drinking and Alcohol Cue-Elicited Neural Activation: A Randomized Trial

Erica N. Grodin, PhD^1^, Spencer Bujarski, PhD^1^, Brandon Towns, BS^1^, Elizabeth Burnette, BS^1,2^, Steven Nieto, PhD^1^, Aaron Lim, MA^1^, Johnny Lin, PhD^3^, Karen Miotto, MD^4^, Artha Gillis, MD, PhD^4^, Michael R. Irwin, MD^1,4,5,6^, Christopher Evans, PhD^4,7^, Lara A. Ray, PhD^1,4,7^

**Supplemental Information:**

**Methods.** Neuroimaging Procedures

**Figure S1.** Whole Brain Activation: Alcohol vs. Beverage Contrast Across All Participants

**Table S1.** Whole Brain Activation: Alcohol vs. Beverage Contrast Across All Participants

**Table S2.** Adverse Events by Medication Condition

**Table S3.** Medication Compliance by Medication Condition

**Results.** Medication Effects on Any Drinking

**Table S4.** Effect of Ibudilast on Any Drinking

**Figure S2**. Medication by Drinking Day Interaction on Self-Reported Craving

**Methods**

***Neuroimaging Procedures***

A T2‐weighted, high‐resolution matched‐bandwidth (MBW) anatomical scan (time to repetition (TR) = 5,000 ms, time to echo (TE) = 34 ms, flip angle = 90°, voxel size: 1.5 mm × 1.5 × 4 mm, field of view (FOV) = 192 mm2, 34 slices, ~1.5 minutes) and a T1‐weighted magnetization‐prepared rapid gradient‐echo (MPRAGE) sequence (TR = 2,530 ms, TE = 1.74 ms, time to inversion = 1,260 ms, flip angle = 7°, voxel size: 1 mm3, FOV = 256 mm2, ~6.2 minutes) were acquired for co‐registration to the functional data. A T2*‐weighted echo planar imaging (EPI) scan (TR = 2,200 ms, TE = 35ms, flip angle = 90°, FOV = 192 mm, slices = 36, 3.0 mm, ~12 minutes) was acquired to examine the blood oxygen-level dependent (BOLD) signal during the alcohol cue reactivity task.

**Figure S1 – Whole Brain Activation: Alcohol vs. Beverage Contrast Across All Participants**

**
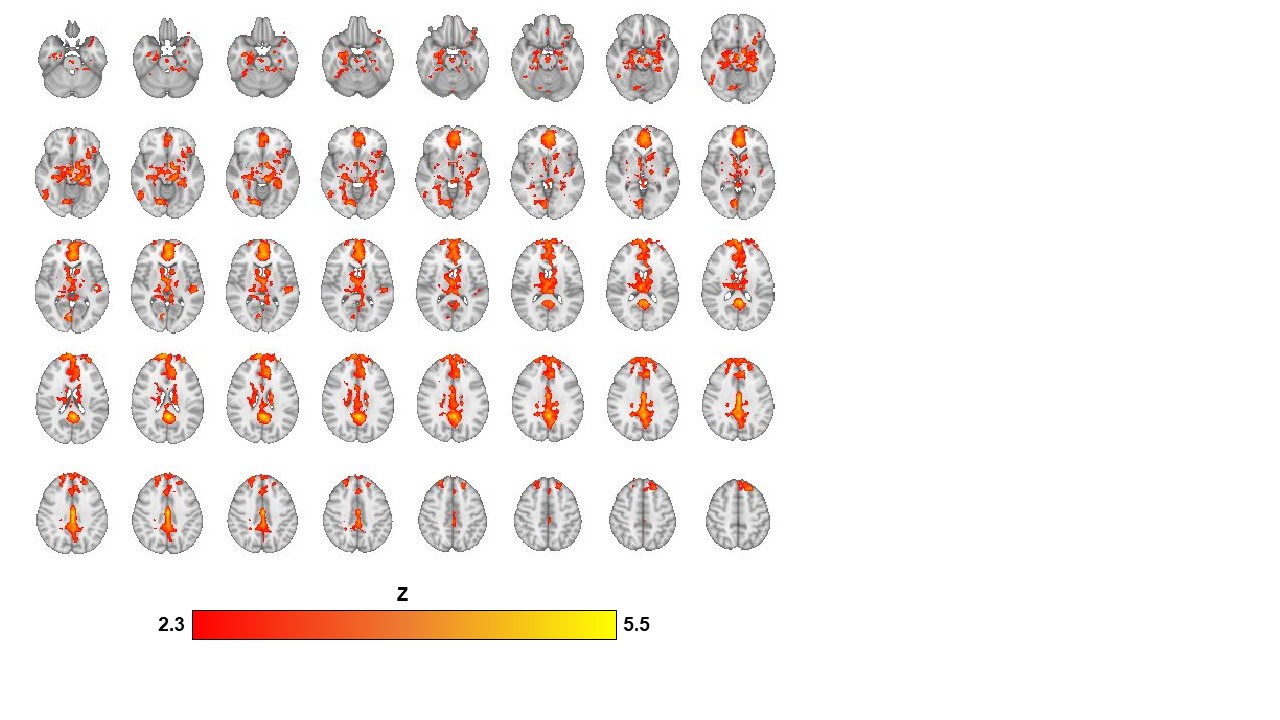
**

Brain activation to alcohol cues across all participants. the fMRI. Z‐statistic maps are whole‐brain cluster corrected, Z > 2.3, p = 0.05. Coordinates are in MNI space. Brain is displayed in radiological convention (L = R).

**Table S1 – Whole Brain Activation: Alcohol vs. Beverage Contrast Across All Participants**

| **Brain Region** | **Cluster Voxels** | **Max Z** | **x** | **y** | **z** | **p-value** |
| --- | --- | --- | --- | --- | --- | --- |
| L Superior Frontal Gyrus | 7,115 | 4.11 | --6 | 14 | 70 | <0.001 |
| Anterior Cingulate Gyrus |  | 3.79 | -8 | 32 | 32 |  |
| Cingulate Gyrus |  | 3.71 | 0 | -16 | 36 |  |
| Posterior Cingulate Gyrus |  | 3.31 | -4 | -48 | 22 |  |
| Medial Prefrontal Cortex |  | 3.03 | -4 | 56 | 2 |  |
| L Insula | 2,126 | 4.18 | -32 | 18 | -12 | <0.001 |
| R Caudate |  | 3.57 | 12 | 2 | 12 |  |
| R Nucleus Accumbens |  | 3.24 | 14 | 16 | -4 |  |
| L Caudate |  | 3.22 | -12 | 10 | 8 |  |
| L Accumbens |  | 2.65 | -12 | 16 | -4 |  |

**Table S2**

Adverse Events by Medication Condition

| Adverse Event | Ibudilast | Placebo | Test for Difference |
| --- | --- | --- | --- |
| **Gastrointestinal** | **10** | **9** | **χ^2^ = .70, *p* = .40** |
| Nausea | 6 | 3 |  |
| Vomiting | 1 | 1 |  |
| Diarrhea | 1 | 0 |  |
| Dyspepsia | 1 | 2 |  |
| Decreased appetite | 0 | 2 |  |
| Frequent bowel  movements | 1 | 0 |  |
| Gut pain | 0 | 1 |  |
| **Nervous System** | **5** | **4** | **Fisher’s Exact Test, *p* = .57** |
| Dizziness | 0 | 1 |  |
| Headache | 1 | 1 |  |
| Disorientation | 1 | 0 |  |
| Vision Blurred | 1 | 0 |  |
| Somnolence | 0 | 1 |  |
| Insomnia | 2 | 1 |  |
| **Psychiatric** | **3** | **7** | **Fisher’s Exact Test, *p* = .49** |
| Libido Increased | 0 | 1 |  |
| Depression | 0 | 3 |  |
| Anxiety | 2 | 1 |  |
| Grief Reaction | 0 | 1 |  |
| Irritability | 1 | 1 |  |
| **Respiratory** | **3** | **2** | **Fisher’s Exact Test, *p* = .56** |
| Cold (Influenza) | 2 | 2 |  |
| Asthmatic Crisis | 1 | 0 |  |
| **General** | **2** | **1** | **Fisher’s Exact Test, *p* = .58** |
| Cold Sweat | 1 | 0 |  |
| Lethargy | 1 | 1 |  |

**Table S3**

**Medication Compliance by Medication Condition**

| Report Type | Ibudilast | Placebo | Test for Difference |
| --- | --- | --- | --- |
| Self-Report (via DDA) | 98.20 ± 4.45 | 99.13 ± 2.30 | T = 0.86, p = 0.40 |
| Pill Count (in person) | 97.05 ± 5.45 | 97.35 ± 3.91 | T = 0.23, p = 0.82 |

**Results - Medication Effects on Any Drinking**

The final model for any drinking included time, medication (IBUD/PLAC), and baseline drinking days. The main effects of time and medication were not significant (see Table S2). The predicted probability of any drinking in the ibudilast group was 59.25% ± 6.78 (95% CI: 47.62, 69.92), whereas the predicted probability of any drinking in the placebo group was 63.63% ± 4.40 (95% CI: 54.65, 71.75).

**Table S4. Effect of Ibudilast on Any Drinking**

|  |  |  | 95% Confidence Limits | |  |  |  | 95% Confidence Limits | |
| --- | --- | --- | --- | --- | --- | --- | --- | --- | --- |
| Model and Predictor Variables | Parameter Estimate | SE | LL | UL | Z | P | Odds Ratio | LL | UL |
| Any Drinking Days |  |  |  |  |  |  |  |  |  |
| Medication (IBUD) | -0.19 | 0.30 | -0.76 | 0.39 | -0.63 | 0.53 | 0.83 | 0.47 | 1.48 |
| Time | -0.03 | 0.02 | -0.08 | 0.01 | -1.55 | 0.12 | 0.97 | 0.92 | 1.01 |
| **Baseline Drinking Days** | **0.09** | **0.03** | **0.04** | **0.14** | **3.46** | **<0.001** | **1.09** | **1.04** | **1.15** |
|  |  |  |  |  |  |  |  |  |  |

**Figure S2 – Medication by Drinking Day Interaction on Self-Reported Craving**


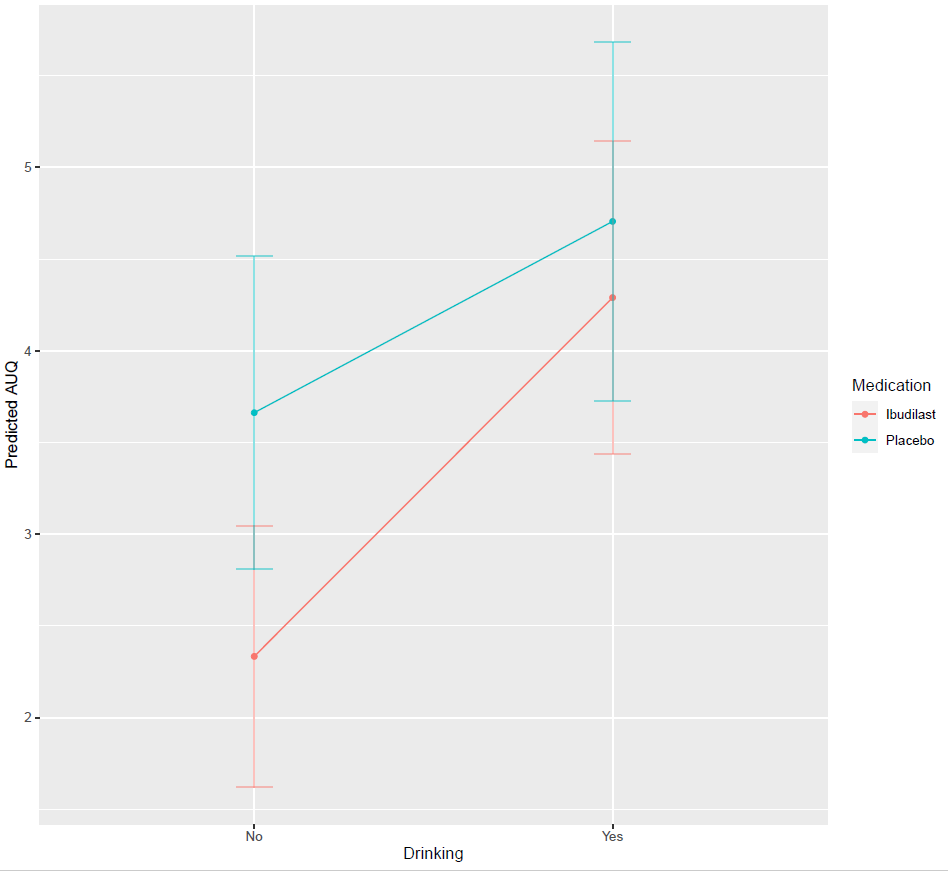

Supplement: Supplementary file 1 — Supplementary Material for Ibudilast, A Neuroimmune Modulator, Reduces Heavy Drinking and Alcohol Cue-Elicited Neural Activation: A Randomized Trial [file 41398_2021_1478_MOESM1_ESM.docx]
